# Development of an improved vaccine for contagious bovine pleuropneumonia: an African perspective on challenges and proposed actions

**DOI:** 10.1186/1297-9716-44-122

**Published:** 2013-12-20

**Authors:** Joerg Jores, Jeffrey C Mariner, Jan Naessens

**Affiliations:** 1 International Livestock Research Institute, Old Naivasha Road, PO Box 30709, 00100 Nairobi, Kenya

## Abstract

Contagious bovine pleuropneumonia (CBPP) caused by *Mycoplasma mycoides* subsp. *mycoides* (*Mmm*) is an economically very important cattle disease in sub-Saharan Africa. CBPP impacts animal health and poverty of livestock-dependent people through decreased animal productivity, reduced food supply, and the cost of control measures. CBPP is a barrier to trade in many African countries and this reduces the value of livestock and the income of many value chain stakeholders. The presence of CBPP also poses a constant threat to CBPP-free countries and creates costs in terms of the measures necessary to ensure the exclusion of disease. This opinion focuses on the biomedical research needed to foster the development of better control measures for CBPP. We suggest that different vaccine development approaches are followed in parallel. Basic immunology studies and systematic OMICs studies will be necessary in order to identify the protective arms of immunity and to shed more light on the pathogenicity mechanisms in CBPP. Moreover a robust challenge model and a close collaboration with African research units will be crucial to foster and implement a new vaccine for the progressive control of this cattle plague.

## Table of contents

1. An African perspective on CBPP

2. What do we know about immunity to infections with *Mycoplasma mycoides* subsp. *mycoides*?

3. A better CBPP challenge model is needed

4. Empirical vaccine approach versus design of a rational vaccine?

5. What knowledge is needed to foster the development of a better vaccine?

5.1 Establish a method to induce solid immunity

5.1 Identify protective host immune responses

5.1 Understand host-pathogen interaction

5.1 Understand the epidemiology of CBPP

6. Research carried out in developed and developing countries

7. Abbreviations

8. Competing interests

9. Author’s contributions

10. Acknowledgements

11. References

## 1. An African perspective on CBPP

**Figure 1:**
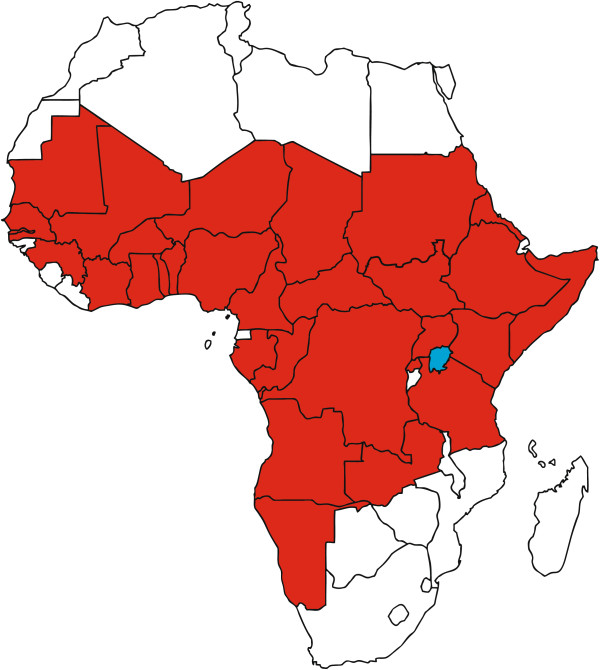
**Occurrence of contagious bovine pleuropneumonia in Africa between 2010 and 2013.** Countries displayed in red have recorded cases of CBPP. All other countries have been free from the disease or no reported data. Data were collected from the website of the World Organization of Animal Health [1].

Contagious bovine pleuropneumonia (CBPP) caused by *Mycoplasma mycoides* subsp. *mycoides* (*Mmm*) was introduced to Africa in the colonial era and subsequently spread throughout the continent. Control efforts prior to independence and in the early post-colonial period were based on stringent movement control combined with vaccination campaigns supplemented by a policy of test and slaughter. These early efforts were successful in suppressing the disease and eventually eradicated CBPP from a number of countries in Southern Africa. Over the last few decades Africa has experienced a resurgence of CBPP and the disease has been reported in many countries of sub-Saharan Africa (Figure 1). Presently control of CBPP relies on a live vaccine of limited efficacy and duration of immunity with occasional severe side effects [2]. OIE-recommended diagnostic tests have limited sensitivity and are primarily useful at herd but not at individual level [3]. A policy of strict movement control and test and slaughter is at this time not possible to implement in most regions because of public resistance, mobile production systems tailored to highly variable rainfall patterns, fragmented veterinary services and lack of funds for compensation [4]. Publicly funded mass vaccination programs have not been sustainable leading to infrequent or sporadic control. Currently, farmers and field veterinarians rely heavily on antimicrobials to reduce the impact of CBPP, although this practice is often not permitted under official policy.

Theoretically, the proper application of current vaccines could contribute to control programs for CBPP in many parts of Africa but offers many practical challenges in terms of the need for near absolute movement control, continuous annual revaccination and surveillance. Mariner et al. [4] created an epidemiological model for a pastoral environment, that included many variables influencing transmission, and concluded that with the current live vaccine and diagnostic tests it would be impossible to eliminate the pathogen from endemic areas given the current socio-economic context. While one can debate the estimates used, the model allows one to gauge the effect of each parameter on prevalence and spread of disease. The current situation, characterized by the spread and reappearance of CBPP in large areas, supports the findings of the modelling. *Mmm* is a recently evolved pathogen with very little sequence variation [5]. Genetic diversity will increase in *Mmm* in case CBPP expands in Africa as observed during the last decades. Because of the latter, urgent development and implementation of better control measures is required.

A better vaccine that protects animals for more than two years, requires only a single injection, does not need a cold chain and is not associated with adverse reactions is key for the progressive control within all regions of the continent as stated recently at an international CBPP workshop [5]. However, new tools alone are not enough. The technological innovations need to be integrated into effective control strategies and service delivery institutions that harness incentives to drive the participation of livestock owners and service delivery actors [6].

## 2. What do we know about immunity to infections with *Mycoplasma mycoides* subsp. *mycoides*?

Experimental findings reported in the CBPP literature are often contradictory and difficult to integrate in a coherent interpretation. Conventional wisdom on CBPP as reported in the text books is often not supported by documented evidence, or not rigorously tested for its accuracy. For example, whilst naturally recovered animals are assumed to be immune, hard evidence from well-designed experiments to test this belief is lacking. It is based on field observations that recovered animals do not become re-infected, but there is no systematic information on what is understood by “infected and recovered”, nor what percentage of recovered cattle would be immune, or for how long.

Injection with the current live vaccine results in protection of up to one year but does not prevent the development of pathomorphological lesions after challenge suggesting induction of limited immunity. Unfortunately, there is no reported method to consistently induce solid immunity against *Mmm* infections or rigorous evidence that such a state in fact exists. The current understanding of CBPP immunology does not allow us to name the main protective mechanism. For instance antibody transfer experiments carried out in the past are difficult to interpret [7], as the amount and specificity of antibodies present in the donor and recipient were not known and total serum, not purified, antibodies was transferred. Different views about the protective role of CD4^+^ T cells in the course of a primary infection were published [8,9]. Therefore it is necessary to carry out basic studies that assess the role of T cells and antibodies from protected animals, such as after vaccination, in an unambiguous way.

## 3. A better CBPP challenge model is needed

A suboptimal challenge model that requires relatively large animal numbers hampers CBPP research. The current intubation model should be replaced by a more robust, uniform challenge model, which would allow the comparison of data from different experimental infections. In-contact infections, which resemble the natural course of infection, do not allow the simultaneous induction of CBPP in all animals to be infected, which limits its value as a tool in comparative trials.

We urge the establishment of a robust uniform challenge bovine model for CBPP (specification of minimal infective dose, host age and breed, and *Mycoplasma* isolates) so that data can be better compared between laboratories. We suggest exploring the use of *Mycoplasma*-containing aerosols for infection since this resembles the natural route of infection best. In parallel we believe that the development of a caprine infection model, which will enable us to test well-targeted mutants of the closely related pathogen *Mycoplasma mycoides* subsp. *capri* will be beneficial for basic proof of concept studies in CBPP. The establishment of both models will be more cost effective and foster CBPP research outcomes worldwide.

## 4. Empirical vaccine approach versus design of a rational vaccine?

The current live vaccines based on the T1 strain have limited efficacy and do only allow improvements though genetic manipulation of the strain or the introduction of alternative media to foster different expression patterns. The T1/44 strain has been reported to be attenuated through 44 passages in eggs [10], but it can still cause disease when applied directly into the lungs [11], the normal route for provoking an experimental infection. The Chinese vaccine strain, BEN-1, which has been applied for the progressive control of CBPP in China [12] requires evaluation in African cattle breeds challenged by African isolates in order to compare its efficacy with the current live vaccine T1/44.

The generation of a genetically modified live vaccine is theoretically possible but implementation of the vaccine in the field would be challenging because of expected delays by biosafety and veterinary regulatory bodies to use such an organism throughout Africa.

Empirical approaches based on individual combinations of antigens or antigen preparations including a bacterin type of vaccine are easy to assess in terms of their efficacy and do not require detailed knowledge on the protective arm of immunity or the pathogenicity mechanisms involved. Their read out is simple: protection or no protection after challenge. But if a combination of individual antigens for incorporation into a subunit vaccine is needed, even *Mycoplasma mycoides* subsp. *mycoides*, with a genome size of about 1 Mb, still offers ample combinations of immunogens to assess in costly challenge experiments without prior selection. Although one could argue that the latter is also a rational approach to vaccine development we suggest reserving the term rational for an approach as described below.

Reverse vaccinology and the “OMICS” era offer tools to narrow down the number of molecules to be tested, through identification of virulence traits using comparative genomics and in vivo proteomics and transcriptomics, and we urge that such an approach is followed in parallel.

Several arguments support the initiation of a rational strategy, despite the fact that systematic, empirical approaches might provide quick answers. The latter are always based on an unproven hypothesis, such as that antibody titres correlate with immunity or that protective antigens are protein in nature. Immunization with a single lipoprotein antigen resulted in enhanced pathology after challenge, suggesting adverse vaccine outcomes [13]. The same was found in experiments for a subunit vaccine to *M. bovis*[14]. These observations indicate that such antigens should be avoided in a vaccine and preference should be given to a (subunit) vaccine consisting of protective antigens only. Similarly, some host responses might exacerbate disease rather than protect. Alternatively, a response to a particular antigen might fail to detect the appropriate epitope that could induce protection, such as the cattle response against GlpO [15]. Up till now, empirical approaches have not delivered the promised vaccine.

## 5. What knowledge is needed to foster the development of a better vaccine?

### 5.1 Establish a method to induce solid immunity

It is desirable to establish a reproducible method to induce solid immunity against *Mycoplasma mycoides* subsp. *mycoides*. Validating the extent and the nature of immunity in fully recovered natural infections or animals immunized with the current live vaccine will be a good starting point. A better challenge model, as discussed above, will help us to read out such an immune state.

### 5.2 Identify protective host immune responses

Generation of solidly immune animals, if possible, will be essential to identify the protective responses that prevent clinical disease. Since there is no rodent model for CBPP, direct experiments on ruminants have to be carried out to derive evidence. Experiments such as antibody transfer, depletion of specific leukocyte subpopulations, neutralization of immune functions or cytokines should be employed to classify particular responses during a course of CBPP. Having a better understanding of the type of responses that provide protection will allow us to design a vaccine via tailored adjuvants and delivery systems that specifically induces those responses. The research community should make an effort to characterize the dynamics of the innate responses during disease since these responses prime subsequent adaptive immune response and might give insight into the pathogenesis during CBPP.

We also suggest looking in more detail into the protective immunity mechanisms of the bacterin type of the vaccine against contagious caprine pleuropneumonia (CCPP), which clinically resembles CBPP. This vaccine has proven to confer immunity [16] against *Mycoplasma capricolum* subsp. *capripneumoniae*, which is also a member of the so called “*Mycoplasma mycoides* cluster” and phylogentically related to *Mmm*[17]. Understanding the protective mechanisms induced by the CCPP vaccine may inform us about the bovine situation.

### 5.3 Understand host-pathogen interaction

Having an understanding of the mechanisms and the sequence of molecular events that lead to disease are important to identify virulence traits and protective antigens. The published sequence of the type strain PG1 [18] was a milestone in CBPP research and enabled subsequent downstream applications such as proteomics [19,20] and reverse vaccinology. Virulence differences in African and European isolates have been reported [21] and enabled the identification of a metabolic enzyme as virulence trait in the past [22].

Systematic OMICs studies such as genomics, transcriptomics, proteomic, lipidomics and glycomics should be carried out and integrated into systems biology approaches in order to better understand the pathogen, the host and their interactions. Preliminary efforts have been made in that direction, such as monitoring the kinetics of antibody specificities using bead assays [20] and the characterization of in vitro surface core proteome [23]. These data are now available and will contribute to detailed information regarding individual antibody responses to help identify protective or pathological responses.

Finally, we need to investigate the nature of host-pathogen interactions in a more systematic way and identify interactions that correlate with disease severity. OMICs data are likely to provide more insight into the molecules that shape pathogenicity, virulence and host specificity. Recently, techniques for the targeted mutagenesis of members of the “*M. mycoides* cluster” have been developed as part of synthetic biology efforts [24] and awaiting their application in CBPP research. In vitro assays such as those that measure adhesion to different cell lines of the respiratory tract and those that examine interactions with host cells such as macrophages have to be established and will allow the screening for molecules that mediate host-pathogen interactions, using targeted mutagenesis.

### 5.4 Understand the epidemiology of CBPP

Better epidemiological data for CBPP are needed in order to integrate the specifications of the current and future control measures (vaccines and diagnostics) in a model that is more robust with less unknown parameters than the current one. Important parameters include the rate of transmission, and the effect of antimicrobial treatments and vaccination on transmission. Such experiments are costly but needed in order to have a coherent epidemiological model which advices policy makers in Africa with respect to decisions that underpin a progressive disease control.

## 6. Research carried out in developed and developing countries

We believe that a partnership of laboratories in developing and developed countries will be essential to tackle CBPP. Research goals will be more easily achieved by performing in vivo experiments in Africa. Past experiments infecting mice are in our opinion of little value, as the mycoplasmas do not induce disease, and are not better than culturing mycoplasma in vitro, offering little promise for the future. The reduced cost of experiments in Africa makes it possible to use larger numbers of animals, while the endemicity of the pathogen reduces the risks associated with pathogen escape into a naïve population. Further, working in endemic areas provides access to the genetic diversity of cattle that are at risk and avoids the need for shipping samples, which may affect measurements from subsequent experiments involving live host cells. Finally, it also builds capacity in the region helping to support laboratories on the continent that will be needed in the future to implement the new products.

## Abbreviations

CBPP: Contagious bovine pleuropneumonia; Mmm: *Mycoplasma mycoides* subsp. *mycoides*; Mmc: *Mycoplasma mycoides* subsp. *Capri* .

## 8. Competing interests

The authors declare that they have no competing interests.

## 9. Authors’ contributions

JJ and JN drafted the manuscript and JM added an epidemiological perspective. All authors read and approved the final manuscript.

## References

[B1] World Animal Health Information Database (WAHID) Interface [http://www.oie.int/wahis_2/public/wahid.php/Wahidhome/Home]

[B2] Thiaucourt F Yaya A Wesonga H Huebschle OJ Tulasne JJ Provost A Contagious bovine pleuropneumonia. A reassessment of the efficacy of vaccines used in Africa Ann N Y Acad Sci 2000 44 71 80 11193704 10.1111/j.1749-6632.2000.tb05276.x

[B3] Marobela-Raborokgwe C Nicholas R Ayling R Bashiruddin JB Comparison of complement fixation test, immunoblotting, indirect ELISA, and competitive ELISA for detecting antibodies to *Mycoplasma mycoides* subspecies *mycoides* small colony (SC) in naturally infected cattle from the 1995 outbreak in Botswana Onderstepoort J Vet Res 2003 44 21 27 12825677

[B4] Mariner JC McDermott J Heesterbeek JA Thomson G Martin SW A model of contagious bovine pleuropneumonia transmission dynamics in East Africa Prev Vet Med 2006 44 55 74 10.1016/j.prevetmed.2005.09.001 16242799

[B5] Ayling R Meeting Report, Contagious Bovine Pleuropneumonia Workshop, ILRI, Addis Ababa IOM Newsletter 2013 44 10

[B6] Mariner JC House JA Mebus CA Sollod AE Chibeu D Jones BA Roeder PL Admassu B van ‘t Klooster GG Rinderpest eradication: appropriate technology and social innovations Science 2012 44 1309 1312 10.1126/science.1223805 22984063

[B7] Masiga WN Roberts DH Kakoma I Rurangirwa FR Passive immunity to contagious bovine pleuropneumonia Res Vet Sci 1975 44 330 332 766133

[B8] Sacchini F Naessens J Awino E Heller M Hlinak A Haider W Sterner-Kock A Jores J A minor role of CD4+ T lymphocytes in the control of a primary infection of cattle with Mycoplasma mycoides subsp. mycoides Vet Res 2011 44 77 10.1186/1297-9716-42-77 21663697 PMC3148206

[B9] Dedieu L Balcer-Rodrigues V Yaya A Hamadou B Cisse O Diallo M Niang M Gamma interferon-producing CD4 T-cells correlate with resistance to *Mycoplasma mycoides* subsp. *mycoides* S.C. infection in cattle Vet Immunol Immunopathol 2005 44 217 233 10.1016/j.vetimm.2005.04.011 15946743

[B10] Sheriff D Piercy SE Experiments with an avianised strain of the organism of contagious bovine pleuropneumonia Vet Rec 1952 44 615 621

[B11] Mbulu RS Tjipura-Zaire G Lelli R Frey J Pilo P Vilei EM Mettler F Nicholas RA Huebschle OJ Contagious bovine pleuropneumonia (CBPP) caused by vaccine strain T1/44 of *Mycoplasma mycoides* subsp. *mycoides* SC Vet Microbiol 2004 44 229 234 10.1016/j.vetmic.2003.11.007 15036531

[B12] Xin J Li Y Nicholas RA Chen C Liu Y Zhang MJ Dong H A history of the prevalence and control of contagious bovine pleuropneumonia in China Vet J 2012 44 166 170 10.1016/j.tvjl.2011.02.011 21439870

[B13] Nicholas RA Ayling RD McAuliffe L Vaccines for Mycoplasma diseases in animals and man J Comp Pathol 2009 44 85 96 10.1016/j.jcpa.2008.08.004 19111314

[B14] Prysliak T van der Merwe J Perez-Casal J Vaccination with recombinant *Mycoplasma bovis* GAPDH results in a strong humoral immune response but does not protect feedlot cattle from an experimental challenge with *M. bovis* Microb Pathog 2013 44 1 8 23246808 10.1016/j.micpath.2012.12.001

[B15] Mulongo MM Frey J Smith K Schnier C Wesonga H Naessens J McKeever D Cattle immunized against the pathogenic l-alpha-glycerol-3-phosphate oxidase of Mycoplasma mycoides subs. mycoides fail to generate neutralizing antibodies and succumb to disease on challenge Vaccine 2013 44 5020 5025 10.1016/j.vaccine.2013.08.100 24035434 PMC3989769

[B16] Nicholas R Churchward C Contagious caprine pleuropneumonia: new aspects of an old disease Transbound Emerg Dis 2012 44 189 196 10.1111/j.1865-1682.2011.01262.x 21951488

[B17] Fischer A Shapiro B Muriuki C Heller M Schnee C Bongcam-Rudloff E Vilei EM Frey J Jores J The origin of the “*Mycoplasma mycoides* cluster” coincides with domestication of ruminants PLoS One 2012 44 e36150 10.1371/journal.pone.0036150 22558362 PMC3338596

[B18] Westberg J Persson A Holmberg A Goesmann A Lundeberg J Johansson KE Pettersson B Uhlen M The genome sequence of *Mycoplasma mycoides* subsp. *mycoides* SC type strain PG1^T^, the causative agent of contagious bovine pleuropneumonia (CBPP) Genome Res 2004 44 221 227 10.1101/gr.1673304 14762060 PMC327097

[B19] Jores J Meens J Buettner FF Linz B Naessens J Gerlach GF Analysis of the immunoproteome of *Mycoplasma mycoides* subsp. *mycoides* small colony type reveals immunogenic homologues to other known virulence traits in related *Mycoplasma* species Vet Immunol Immunopathol 2009 44 238 245 10.1016/j.vetimm.2009.04.016 19443045

[B20] Hamsten C Neiman M Schwenk JM Hamsten M March JB Persson A Recombinant surface proteomics as a tool to analyze humoral immune responses in bovines infected by *Mycoplasma mycoides* subsp. *mycoides* small colony type Mol Cell Proteomics 2009 44 2544 2554 10.1074/mcp.M900009-MCP200 19696080 PMC2773720

[B21] Abdo el-M Nicolet J Miserez R Goncalves R Regalla J Griot C Bensaide A Krampe M Frey J Humoral and bronchial immune responses in cattle experimentally infected with *Mycoplasma mycoides* subsp. *mycoides* small colony type Vet Microbiol 1998 44 109 122 10.1016/S0378-1135(97)00184-3 9549852

[B22] Vilei EM Frey J Genetic and biochemical characterization of glycerol uptake in *Mycoplasma mycoides* subsp. *mycoides* SC: its impact on H(2)O(2) production and virulence Clin Diagn Lab Immunol 2001 44 85 92 11139200 10.1128/CDLI.8.1.85-92.2001 PMC96015

[B23] Krasteva I Liljander A Fischer A Inglis NF Smith DG Scacchia M Pini A Jores J Sacchini F Characterization of the *in vitro* surface core proteome of *Mycoplasma mycoides* subsp. *mycoides*, the causative agent of contagious bovine pleuropneumonia Vet Microbiol 2014 44 116 123 10.1016/j.vetmic.2013.10.025 24332827

[B24] Lartigue C Vashee S Algire MA Chuang RY Benders GA Ma L Noskov VN Denisova EA Gibson DG Assad-Garcia N Alperovich N Thomas DW Merryman C Hutchison CA 3rd Smith HO Venter JC Glass JI Creating bacterial strains from genomes that have been cloned and engineered in yeast Science 2009 44 1693 1696 10.1126/science.1173759 19696314

